# Kelp forests versus urchin barrens: a comparison of ecosystem functions and services provided by two alternative stable marine habitats

**DOI:** 10.1098/rspb.2024.1539

**Published:** 2024-11-06

**Authors:** Aaron M. Eger, Caitlin O. Blain, Amelia L. Brown, Sharon S. W. Chan, Kelsey I. Miller, Adriana Vergés

**Affiliations:** ^1^ Center for Marine Science and Innovation, University of New South Wales, Sydney 2052, Australia; ^2^ Kelp Forest Alliance, Sydney 2034, Australia; ^3^ Leigh Marine Laboratory, Institute of Marine Science, University of Auckland, Leigh 0985, New Zealand; ^4^ Coastal People Southern Skies Centre of Research Excellence, University of Otago, Dunedin, New Zealand

**Keywords:** kelp forest, urchin barren, ecosystem function, alternative stable states, phase shift

## Abstract

Kelp forests and urchin barrens are two stable states in rocky reef ecosystems, each providing unique ecosystem functions like habitat for marine species and primary production. While studies frequently show that kelp forests support higher levels of some ecosystem functions than urchin barren habitats, no research has yet compared average differences. To address this gap, we first conducted a meta-analysis of studies that directly compared the ecosystem functions, services and general attributes provided by each habitat. We also compiled individual studies on ecosystem properties from both habitats and qualitatively assessed the benefits provided. The meta-analysis included 388 observations from 55 studies across 14 countries. We found that kelp forests consistently delivered higher levels of ecosystem properties such as biodiversity, species richness, abalone abundance and sea urchin roe quality. Urchin barrens supported higher urchin density and crustose coralline algae cover. The qualitative review further supported these findings, showing that kelp forests ranked higher in 11 out of 15 ecosystem properties. These findings can help guide decisions on managing rocky reef habitats and demonstrate the benefits of preserving or expanding kelp forests.

## 1. Introduction

Terrestrial and marine ecosystems often exhibit alternative stable states [1,2], wherein changes in a key element of the system result in a dramatic and lasting shift in species composition and ecosystem functioning [3,4]. Historically, these ‘regime shifts’ would result in the alternative states shifting over extended periods of time depending on the persisting environmental conditions [5]. However, there is now also ample evidence that human impacts, such as pollution, climate change, agriculture and overexploitation are key factors inducing regime shifts [6]. For instance, in coral reefs, overfishing and nutrient pollution have led to shifts from coral-dominated to algae-dominated states [7]. These shifts can span large areas (100s of km^2^) and feedback mechanisms can stabilize each state, making it difficult to revert to a previous state, a process known as hysteresis [8,9]. Hysteresis means that the tipping point between stable states differs depending on the direction of the shift. For instance, a vegetated system might become unvegetated with 5 herbivores m^−2^ but require a much lower density of 1 herbivore m^–2^ to return to a vegetated state. This concept is critical for ecosystem management, as it underscores both the technical difficulties in reversing detrimental shifts and the societal challenges in making informed management decisions.

Kelp forests and sea urchin barrens are well-documented examples of alternative stable states in shallow water temperate reef ecosystems [10,11]. Kelp forest ecosystems are dominated by highly productive brown macroalgae (orders Laminariales and Fucales) and form dense, three-dimensional canopies, which provide habitat and food for a variety of species and alter the biophysical characteristics of the marine environment [12,13]. Thousands of fish and invertebrate species live in kelp forests [12,14] and the market-based ecosystem services generated by these systems are estimated at over $500 billion yr^−1^, globally [15]. Urchin barrens are rocky reef habitats that have been formed by high grazing pressure from sea urchins (class Echinoidea), often compounded by other stressors, most notably overfishing of urchin predators [16], storms [17], pollution [18] or increased temperature [19]. As a result, these barrens typically contain very little or no kelp but instead are dominated by crustose coralline algae (CCA) or small, turf-like algae and filamentous algae communities, with significantly less habitat complexity [20]. While these habitats are often considered ‘marine deserts’ [21] or a ‘collapsed state’ of kelp forests [20], they can be home to unique algal species [22], micro-invertebrates [23] and are often important hunting grounds for fish and invertebrate species [24]. Importantly, temperate coastlines often have some mix of kelp forest and urchin barren habitats and this ratio changes over time [20,25]. Though individual studies have typically shown that kelp forest habitats are more productive, biodiverse and provide more highly valued ecosystem services than urchin barrens [26–29], there have been local examples (e.g. New South Wales, Australia) which found unique species living in urchin barren habitats [30].

Humans have influenced the ratio of kelp forests and urchin barren habitats since the late Holocene. For example, traditional hunting of sea otters indirectly impacted sea urchin and kelp forest populations on the West Coast of North America for thousands of years [31] and cod, an Atlantic sea urchin predator, has been harvested for at least a millennium [32]. While human influence on these habitats was typically localized during pre-European colonization, the impact has grown and is now global and large scale [11,20]. Indeed, encroachment by sea urchin populations is a key driver of kelp forest collapse around the world [28,33,34] and the most common cause of failure in kelp forest conservation projects [35]. These regime shifts are driven by an increase in the sea urchin population or grazing rate, which are often triggered by the loss of top predators (i.e. trophic cascades [36]), a recruitment pulse [37] and/or increased range expansion owing to warmer sea temperatures [38]).

As kelp forest habitats around the world decline and urchin barrens increase, interest in intervening to reverse this shift is increasing [35,39,40]. To account for the hysteresis effect, sea urchin density must be greatly reduced (e.g. approx. 10% of the biomass that caused the shift to barrens) for ecosystems to shift to a kelp-dominated state [11]. Efforts to restore kelp forests have focused on controlling sea urchin populations via culling, translocation, fisheries or ecosystem-based management (e.g. restoring top predators [41–43]). These actions [44] seek to turn a system back to a previous historic baseline [44] or to increase ecosystem functioning and the associated services that may benefit human populations [45].

Determining the historical state of marine ecosystems is a key challenge in most regions, as underwater habitat maps are often limited to the past 50–70 years, a much shorter timescale when compared to historical increases in fishing, European expansion and other industrial activities [46]. Instead of making decisions based on historical versus current states, which are often difficult to ascertain, it may be beneficial to quantify the characteristics and benefits of these two associated, alternative and inherently different types of marine ecosystems to inform future marine management of temperate reefs. While many previous studies suggest that kelp forests may provide more services than urchin barrens [10,23,27,47], there has, to our knowledge, been no comprehensive, global comparison of the ecosystem functions or services provided by these two ecosystems to date.

In this study, we catalogue, quantify and compare the different ecosystem services, and thus benefits provided by kelp forest and urchin barren ecosystems globally. We conducted a meta-analysis of the available scientific literature that described an ecosystem property (e.g. biomass, biodiversity and productivity) in paired kelp forests and urchin barren habitats. As there were important ecosystem services that did not have paired studies, we also conducted a qualitative review of select relevant services that are of particular importance to society. These reviews describe the variety of properties provided by these ecosystems and quantify the differences in services provided by kelp forests or sea urchin barrens. This work aims to provide the first comprehensive consolidated evaluation of ecosystem attributes, functions and services in two alternative states and to support informed future management decisions for temperate coastal ecosystems.

## 2. Methods

### (a) Literature search and selection criteria

Our systematic literature search looked for studies that had a paired design and compared ecosystem services or functions between kelp forests (orders Laminariales and Fucales) and urchin barren habitats while controlling for extraneous variables. Because there is no formal definition of the demarcation between a kelp forest and an urchin barren, author definitions were used to make this distinction. We exclusively focused on barren habitat that was sea urchin dominated and did not include other alternative habitats, such as turf or filamentous algae unless urchins were also highly abundant [48].

We included any ecosystem function, service or attribute [49] that was quantifiable between the two habitats and was directional, i.e. where a higher value would be considered a more desired state. Ecosystem functions are the processes and interactions that control the flow of energy and materials through the biotic and abiotic components of an ecosystem, such as primary production or nutrient cycling. Ecosystem services are the benefits humans derive from ecosystems, such as provisioning or regulating services. Ecosystem attributes are the biological and physical characteristics of ecosystems that influence their functions and services, such as taxonomic richness, density or nutritional quality. For simplicity, here we refer to the combination of ecosystem attributes and functions as ‘ecosystem properties’.

We used the comprehensive and traceable search engine Web of Science. We constructed our search terms to encompass the following range of relevant ecological metrics:

— kelp OR seaweed OR macroalga*
— AND
— diversity OR biomass OR product* OR ‘ecosystem services’ OR benefits OR function OR ‘nutrient cycl’ OR ‘cultural services’ OR ‘species richness’ OR recruitment OR nursery OR flux OR scour* OR structure OR abundance
— AND
— urchin OR barren OR CCA OR turf OR coralline flats* OR rock flats* OR grazed OR rocky reef

We first read the titles and abstracts of the returned search results and flagged potential papers for inclusion. We then read each paper in full and identified the ones that met the inclusion criteria for our meta-analysis.

The inclusion criteria required that studies compare an ecosystem attribute, service or function between the two habitats, with study sites in proximity and free from confounding extraneous variables (e.g. habitat protection for one habitat only). The most common reasons for excluding studies include differences in environmental variables between the kelp site and the urchin barren site, such as depth (where the difference between sites was >5 m), wave exposure, abiotic variables (salinity and temperature) or differences between habitats in experimental manipulations. We also required a distinct kelp site and a distinct urchin site and did not include studies that recorded the progression of a single site from kelp forest to urchin barren over time or vice versa. We used a PRISMA diagram to detail the selection process for the papers we included (electronic supplementary material, appendix S1).

### (b) Data extraction and synthesis

If a paper met our inclusion criteria, we then extracted the data on the relevant ecosystem functions or services. We recorded each study site as a unique data point, preserving the individual characteristics and results of each location without averaging across sites. We recorded data separately from the same time and place if variables were independent (e.g. feeding rate and abundance), but did not collect data on variables that were strongly correlated (e.g. biomass and abundance) to avoid redundancy.

Together, data from 129 time and place observations from 55 studies met our criteria. These data represented studies from 14 countries, 11 taxa groups related to the ecosystem property, 22 genera of kelp and 12 different ecosystem properties.

### (c) Statistical approach

We used the escalc function within the metafor package [50] in R to calculate the effect sizes for each unique observation. Since we expected different results, we separated the sea urchin data and CCA from all other taxa and analysed and presented the three sets separately. We then calculated the overall effect sizes across select categories using the lmer function in the lme4 package [51] and removed outlier effect sizes greater than 10 (*n* = 2). In these models, we accounted for the nested structure of our dataset by including the time and date observation number as the mixed effect value. Specifically, we calculated the effect sizes and associated error for two categories: the ecosystem property (e.g. biomass, abundance and productivity; electronic supplementary material, table S1) and the taxonomic grouping (e.g. fish, gastropod and algae; electronic supplementary material, table S2), as well as the interaction between these two categories (e.g. the average biomass for fishes).

We recorded data so that positive values of ecosystem properties corresponded to more ‘desirable’ states (e.g. biomass, density and richness). Therefore, positive effect sizes indicate more of an ecosystem property and thus a more ‘desired’ alternative state in kelp forests and negative effect sizes indicate a more ‘desired’ alternative state in an urchin barren. We only present the results for the taxonomic groups or ecosystem properties that had a sample size greater than two.

### (d) Supplemental literature search and qualitative review

A meta-analysis is the gold standard for comparing effect sizes because it requires a paired comparison between a control and a treatment. This pairing reduces the effect of extraneous environmental and sampling variables and understands the true strength of the effect reported: i.e. the difference in ecosystem services. However, these requirements excluded studies that quantified ecosystem services in kelp forests or urchin barrens individually, which can inform the discussion and evidence base around the topic. We, therefore, conducted a second literature search that removed the paired study requirement and looked for papers that synthesized evidence or quantified values in either ecosystem.

We first selected ecosystem services relevant to kelp forests from the Common International Classification of Ecosystem Services (CICES) list of ecosystem services. We ran a second search, also using Web of Science, targeting the selected ecosystem services (search terms in appendix S2 and results returned in appendix S3) but were not part of a paired study design as required for the meta-analysis. Whenever possible, we focused on studies that presented a body of evidence as opposed to individual data points from a single time or place.

We compiled the relevant studies for each ecosystem service and made a qualitative judgement about whether the service was higher in kelp forests, slightly higher in kelp forests, neutral, slightly higher in urchin barrens or higher in urchin barrens (table 1).

**Table 1.** Results of a qualitative review of ecosystem services provided by kelp forests and urchin barrens. (References in italics were identified by the authors, outside of the structured literature search.) * denotes a search terms wild card (allows for different endings to the same prefix).

ecosystem service	search phrase	comparison	kelp reference	kelp description	kelp value	scale	barren reference	barren description	barren value
seaweed aquaculture	aquaculture	higher kelp	*FAO* [52]	kelp aquaculture is dependent on wild kelp forests for breeding stock	17.48 M tonnes yr^−1^	NA	Angwin *et al*. [53], Pert *et al*. [54], FAO [52]	sea urchins can be used in aquaculture. However, poor quality sea urchins must be fed to enhance their roe and their value as food	0.24 M tonnes yr^−1^
wild harvest	harvest	higher kelp	FAO [52], *Smale et al*. [55], *Forsythe* [56], *Bixler & Porse* [57]	kelp (Fucales/Laminariales) were wild harvested in 17 countries in 2021	7 71 297 tonnes yr^−1^	global	Yoshimoro *et al*. [58]	CCA are not harvested. The rise of urchin barrens negatively affects kelp harvest	NA
wild fisheries	fishery	higher kelp	*Eger et al*. [15]	key fisheries around the world, including lobster and abalone depend on kelp forest habitat	2380 kg ha yr^−1^	global	Angwin *et al*. [53]	sea urchins can be collected from barrens, though they are often roe depleted and require enhancement before they are sellable	NA
nutrient absorption	bioremediation*	not tested	Gaylard *et al*. [59], Eger *et al*. [15]	waste treatment/water purification, including nitrogen removal	$17 608 ha^−1^ yr^−1^ [59]; nitrogen removal - $73 800 657 kg N ha^−1^ yr^−1^) [15]	Australia, global	NA	not present in barrens	NA
nutrient absorption	nutrient cycle	not tested	*Pfister et al*. [60], *Bayley et al*. [61]	kelp beds deplete nitrate and phosphorus concentrations but enhance ammonium and DOC	£2400 M yr^−1^	UK, global	NA	not present in barrens	NA
wave dampening	erosion	higher kelp	Rooijen & Winter [62]	the presence of kelp may significantly affect coastal stability during storm events (modelled study)	NA	global	NA	not present in barrens	NA
wave dampening	wave reduce	higher kelp	Elsmore *et al*. [63], Gaylord *et al*. [64], Mork [65], Jackson & Winant [66], Morris *et al*. [67]	canopy kelp forests have limited, but measurable, capacity to enhance shoreline protection from nearshore waves	NA	global	NA	not present in barrens	NA
fish nursery	nursery	slightly higher kelp	*Holbrook et al*. 1990, *Lazzari* [68], *Olson et al*. [69], *Lefcheck et al*. [70]	kelp forests serve as important nursery habitats for ecologically and economically valuable fishes (e.g. mackerel, cod and rockfish) and invertebrates (e.g. lobster and abalone), though there are some exceptions	NA	NA	*Courtois de Viçose et al*. [71], Takami *et al*. [72]	urchin barrens are sometimes important for the settlement and juvenile growth phase of abalone	NA
pH regulation	pH	higher kelp	Kozal *et al*. [73], Hoshijima *et al*. [74], Ling *et al*. [75], Krause-Jensen *et al*. [76]	the chemical environment within kelp forests positively impacts calcifying organisms, acting as refugia from ocean acidification	NA	global	Ling *et al*. [75]	lower pH overall and less oscillatory	NA
carbon cycle	net primary production	higher kelp	*Pessarrodona et al*. [77]	net primary production	536 g C m^−2^ yr^−1^	global	Pessarrodona *et al*. [77]	net primary production	207 g C m^−2^ yr^−1^
SCUBA/snorkel	recreation	higher kelp	*Lucrezi* [78], *Blamey & Bolton* [79], *Lucrezi et al*. [80]	recreational divers interested in kelp monitoring and citizen science for purposes including: exploration and absorption of wildlife, biodiversity and learning more about kelp ecology	approx. $197.2 M yr^−1^	global	*Hynes et al*. [81]	no evidence of tourism based on barrens (but see Beaumont *et al*. [82]	NA
traditional ecological knowledge (TEK) related to kelp	traditional ecological knowledge	higher kelp	*Thurstan et al*. [83], *Narr* [84], Hurd *et al*. [85]	kelp has been documented as a food source, recreational item, tool and material used in art across multiple traditional and indigenous cultures	NA	NA	NA	there is no available evidence to suggest urchin barrens support traditional ecological knowledge or indigenous cultural practices	NA
kelp ecology courses	education	slightly higher kelp	*Lucrezi* [78], *Blamey & Bolton* [79], *Vasquez et al*. [86]	kelp holds high value as a source of scientific and applied research. Recreational divers interested in kelp monitoring and citizen science	$25 957 253	Chile	NA	urchin barrens are a component of marine ecology courses	NA
kelp arts, movies, etc	art	higher kelp	Hurd *et al*. [85], *Verges et al*. [87]	artistic practices involving kelp (sculpture/painting), photography and shell-stringing culturally important necklaces from species found in seaweed	NA	NA	NA	no evidence for art from barrens	NA
habitat	habitat	higher kelp	Castorani *et al*. [88], *Smale et al*. [55]. *Bologna & Steneck* [89]	see results of this study. Loss of kelp reduces net primary production, detrital flow, mobile invertebrate populations, fish and sessile invertebrate richness, but increases benthic light availability, sub-canopy algae species cover and richness, and sessile invertebrate cover	NA	NA	Castorani *et al*. [88]	see results of this study. Sponge species richness was higher in Cystoseira canopies than in barrens, though species specific as some sponges did better in barrens	NA

## 3. Results

### (a) Meta-analysis

The overall effect size, 0.21 (s.e. = 0.05), indicates that across all properties and taxa, ecosystem properties are significantly higher in kelp forest ecosystems than in urchin barren ecosystems.

### (b) By ecosystem property

Owing to anticipated inherent differences in ecosystem properties between sea urchins, CCA and all other taxa, the effect sizes related to sea urchins and CCA are presented separately.

**Figure 1.**
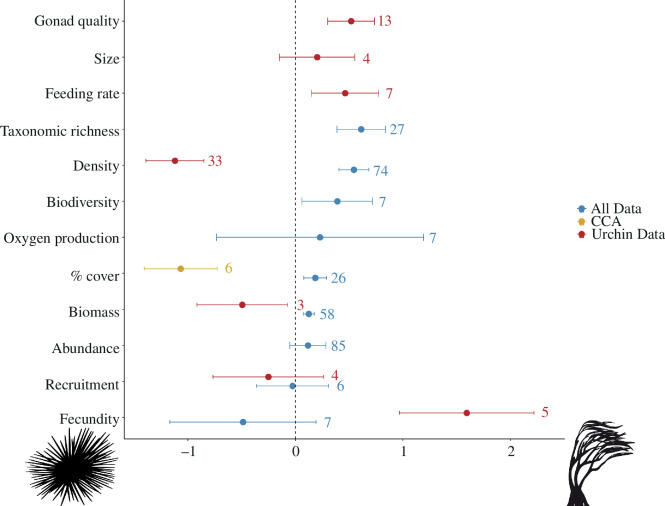
Effect sizes for different ecosystem properties. Data for sea urchins are shown in red, crustose coralline algae (CCA) in yellow and all other data in blue. The dots represent the mean, the error bars represent the standard error and the numbers represent the sample sizes. Values above 0 (to the right) indicate an ecosystem property that is greater in a kelp forest than in an urchin barren, and vice versa for values below 0 (to the left).

We found significant positive effect sizes, or greater properties in kelp forests, across all taxa, excluding sea urchins and CCA, for five of the nine ecosystem properties. These included taxonomic richness (effect size = 0.61), density (effect size = 0.54), biodiversity metrics (effect size = 0.39), per cent cover (effect size = 0.18) and biomass (effect size = 0.12). The other four properties (oxygen production, abundance, recruitment and fecundity) had effect sizes with error bounds across zero and indicated non-significant differences. No properties were significantly higher in urchin barrens for taxa other than sea urchins or CCA (figure 1).

### (c) Ecosystem properties by taxa

For sea urchins, we found significant effects for five of the seven properties. Density and biomass were negative meaning these were significantly higher in barren habitats. However, fecundity, roe quality and feeding rate were higher in kelp forest habitats. For the other two properties, size and recruitment, the differences were not significant.

**Figure 2.**
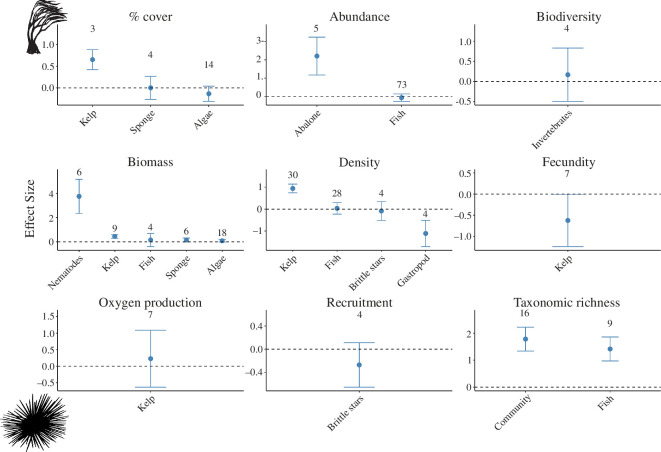
Effect sizes separated by different properties and different taxonomic groupings. All presented data exclude properties related to sea urchins and crustose coralline algae (CCA). The dots show the mean, the error bars show the standard error and the numbers show the sample sizes. Values above 0 (to the right) indicate an ecosystem property that is greater in a kelp forest than in an urchin barren, and vice versa for values below 0 (to the left).

While most combinations of taxa and properties had non-significant effect sizes, there were a few notable exceptions (figure 2). The taxonomic richness of fish communities was significantly higher in kelp forests (effect size = 1.41), whereas the biomass, density and abundance of fishes surveyed had no significant differences. The density (effect size = 0.94), per cent cover (effect size = 0.65) and biomass (effect size = 0.44) of kelp were significantly higher in kelp forests, whereas the oxygen production and fecundity showed no significant differences.

Nine taxa groups only recorded one or two properties per group. Three of these, overall biodiversity (taxonomic richness effect size = 1.79), abalone (abundance effect size = 2.20) and nematodes (biomass effect size = 3.75), were significantly positive, while gastropods, excluding abalone (density effect size = −1.11) and CCA (per cent cover effect size = −0.82) were significantly negative. Properties recorded for algae (biomass and per cent cover), brittle stars (density and recruitment), sponges (biomass and per cent cover effect size) and all other invertebrates (biodiversity) were not significant.

### (d) Qualitative review

We identified 15 additional ecosystem services potentially provided by kelp forests and/or urchin barrens based on CICES. Of these 15 services, 11 were notably higher in kelp forests than in urchin barrens, two were slightly higher in kelp forests and two were not tested (table 1).

## 4. Discussion

Our meta-analysis results show that kelp forests generate more ecosystem functions and higher properties than urchin barrens, including higher biodiversity, taxonomic richness, density, biomass and per cent cover. The only attributes that were higher in urchin barrens were those that inherently define urchin barrens (e.g. sea urchin density and biomass and CCA cover). Importantly, many of the ecosystem properties that were significantly higher in kelp forests are those which are most valued by people: e.g. taxonomic richness, abalone density and roe quality of sea urchins. We did not find significant variation across kelp genera or locality, indicating that these results are generalizable to kelp–urchin barren systems worldwide (electronic supplementary material, figures S1 and S2). In the first comprehensive review (to our knowledge) we show that overall, kelp forests provide higher levels of multiple ecosystem attributes, functions and services than urchin barrens.

Kelp forests and urchin barrens will always be co-occurring temperate reef ecosystems that support unique and shared biodiversity and ecosystem properties. As more kelp forests shift into and remain urchin barrens, managers can use these results to make informed decisions when deciding if kelp restoration is appropriate.

### (a) Ecosystem properties higher in kelp forests

This study showed that kelp forests, overall, have higher levels of multiple ecosystem properties than urchin barrens (effect size = 0.21). These included several keystone components of temperate marine ecosystems, including taxonomic richness, biodiversity metrics, macroalgae biomass and per cent cover [90]. In addition, several properties related to sea urchins: fecundity, roe quality and feeding rates also had higher values in kelp forests. Therefore, while the density and biomass of urchins are higher in barrens, urchin health and their value as a prey and fishery species are the highest when living in kelp forests [27,91]. In addition to underpinning the function of healthy temperate reef ecosystems, these functions also underpin the ecosystem services that are most valued by people. For instance, the evidence suggests that many more adult abalone live in kelp forests compared to sea urchin barrens (effect size = 2.20). Furthermore, properties like habitat structure, biodiversity and secondary productivity are linked to key services and human activities, such as snorkelling, SCUBA diving, commercial fisheries, recreational fisheries and cultural practices [92,93].

### (b) Ecosystem attributes higher in urchin barrens

The only attributes higher in urchin barrens were those that are intrinsically linked to those ecosystems, namely sea urchin biomass and density and per cent cover of CCA, yet these features do not equate to greater ecosystem services. While barren-forming sea urchins can be a prized fishery, our results show that while the population density and total biomass are higher, urchins in barrens have lower quality roe and are less fecund, which reduce fisheries yield [94] and can limit their nutritional value to predators [95]. Furthermore, the higher per cent cover of CCA is frequently attributed to overgrazing of other forms of erect algae, e.g. more nutritious turfs and macroalgae [20,96]. CCA support a specific set of invertebrates [97] and are associated with settlement cues for different marine species [98]. Net biodiversity and taxonomic richness were highest in kelp forests. However, there was high variability among some studies and two taxa, gastropods (density) and sea urchins (density and biomass), had significantly greater values within barrens.

### (c) Ecosystem properties with no difference between the two ecosystems

Many effect sizes did not show significant differences between the two ecosystems. For fishes, taxonomic richness was significantly different, whereas biomass, density and abundance were not. These results illustrate that the effect of habitat on ecosystem properties may vary across taxa and that the properties within each taxon may also vary. For instance, species that require low complexity habitat for settlement, breeding or hunting, e.g. limpets (*Cellana* spp.), top-shells, *Patelloida* and *Australium* spp. [99] and the fishes, damselfishes *Parma microlepis* [100] and *Hypoplectrodes maccullochi* [101] are more abundant or are at least more conspicuous in barrens in Australian temperate reefs. By contrast, species that require more habitat structure and kelp for growth, e.g. abalone (*Haliotis* spp.) [102] and rockfish (*Sebastes* spp.) prefer kelp forests [103]. In biodiversity assessments, while more species overall inhabit kelp forests, each habitat can host unique assemblages and species not observed in the other [10,104]. Therefore, the highest biodiversity at the seascape level is sometimes found when there is a mosaic of habitats.

We found non-significant results for other properties, including algae biomass and per cent cover, oxygen production, body size and recruitment. However, given the small sample sizes involved, many of these results had high levels of variance and it is difficult to make firm conclusions without additional data. The results on oxygen production and kelp fecundity may be surprising, as these are frequently cited as benefits of kelp forests. Other studies have demonstrated the high primary productivity of kelp forests relative to algal turfs or CCA [47,105] and higher fecundity should be inherently higher overall in a kelp forest versus an urchin barren. For both results, data originated from a single study each [106], excluded large macroalgae from being measured in benthic chambers and found variable rates of oxygen production at the site level and non-significant differences between kelp forests and barren habitats. Edwards & Konar [107] found that per capita kelp fecundity was higher in urchin barrens, as the few remaining sporophytes received more sunlight, nutrients and current. However, this finding does not imply that the net fecundity of an area is higher in barrens. These contradictions highlight the limits of meta-analyses when sample sizes are small and high variances are involved. Further research will allow more accurate and general conclusions about these functions. Several studies reported ecosystem properties in only one ecosystem [108,109] and future research should use a paired design with kelp and urchin barrens to allow us to understand the counterfactual, i.e. what are the functions gained or lost when one system flips to another.

### (d) Qualitative study results

Of the 15 ecosystem services identified from the CICES framework, 11 were higher in kelp forests, two were slightly higher in kelp forests, two were not evaluated and none were higher in urchin barrens. This review identified several services that were not found to be directly compared in the meta-analysis.

Notably, the net primary production of kelp forests globally was over two times higher than CCA beds that characterize urchin barrens. While not all services were reported for urchin barrens, these higher rates of productivity intrinsically mean that kelp forests support more ecosystem services through their photosynthetic capacity, including oxygen production, pH modulation, nutrient remediation and carbon sequestration. Marine carbon dioxide removal is a key service of interest to modern policy [110,111]. In kelp forests, carbon dioxide removal involves a combination of both dissolved organic carbon and particulate organic carbon. Conversely, CCA habitat mostly contributes to carbon dioxide removal via dissolved organic carbon. While carbon dioxide removal in CCA beds is not well studied [112], it is probably less than half that of kelp given the absence of a particulate organic carbon pathway. Further quantification of the level of services provided by urchin barrens will help better understand the difference between the two habitats.

The enhanced biological structure and biomass of kelp forests also mean that they support wild seaweed harvest industries while barrens do not. Similarly, kelps are commonly aquacultured (17.8 M tonnes in 2021), while CCA is not. Kelp forests on average support 2.4 tonnes of fisheries biomass per hectare each year, though comparable numbers are not available for urchin barrens or CCA beds. Indeed, as highlighted in this study, some of that kelp-dependent biomass is from prized species, such as abalone [92], lobster [113] and sports fishes [15]. A key service provided by urchin barrens is a nursery habitat, notably for the early life stages of some abalone that often settle on and develop in more exposed CCA habitats before moving to denser kelp forests [72].

Cultural services such as tourism, art, traditional ecological knowledge [85] and education were also all presumably higher in kelp with little to no evidence to suggest those services are provided by barrens. Indeed, there are art exhibits, food festivals and community events centred around kelp forests [80]; festivals linked to urchin consumption similarly exist, notably in Japan [114]. While barrens host sea urchins, urchins have higher roe quality and are more desirable in kelp forests. There was little evidence suggesting urchin barrens support social activity and well-being. This qualitative review complements the quantitative meta-analysis and supports the conclusion that kelp forests provide more services than urchin barrens.

### (e) Implications for marine management

Understanding which ecosystem properties are indeed higher in kelp forests helps guide decisions for ecosystem management and set realistic expectations. These results can guide information-based decision-making around protecting or restoring kelp forests and help inform benefit–cost decisions in marine management. Historically, restoration has been motivated by a desire to restore ecosystem functions [115,116] and the results of this study support those assumptions and can help justify similar projects. Notably, these results should advance the benefit–cost analyses that inform decision-making in ecosystem restoration [117].

Alternative stable states present management decisions about what level of intervention is required or desired to benefit people or ecosystems [118,119]. Society often considers restoration most justified if there is clear evidence that the reef area was previously a kelp forest and shifted to urchin barren owing to anthropogenic stressors, such as overfishing [16]. This scenario is established within the paradigm of ‘ecological restoration’ [120]. However, there is often equivocal evidence or no data on the historical balance between urchin barrens and kelp forests, and managers will need to use other information to make that decision. Managers may wish to make that decision based on the cost–benefit analysis of increasing ecosystem services versus sea urchin removal [30,101,120]. These results can help inform the outcome of these decisions and provide a case study for managing other similar system interactions.

Importantly, these findings also contribute to answering the question of what happens when a system changes from kelp forest to urchin barren. While past studies focused on the functions or services related to kelp forests [15,79,86,93], the meta-analysis approach provides the counterfactual. This counterfactual is needed to perform more accurate economic evaluations [121] and guide benefit–cost decision-making [122]. These benefits will be particularly useful in the emerging field of environmental [123] and ocean accounting [124].

Finally, managers must always consider the prospects for success in converting urchin barrens into kelp forests. The standard in restoration is to only attempt to restore an ecosystem once the initial cause of decline has been removed or mitigated [120]. Understanding the cause(s) of kelp loss and drivers of the expansion of sea urchin barrens (e.g. predator loss, elevated sea temperatures, pollution and invasive species) remains critical for developing effective restoration plans. However attractive it may be to restore a kelp forest based on the described benefits, managers need to address these root causes or risk wasting their efforts.

### (f) Expanding the evidence base

**Figure 3.**
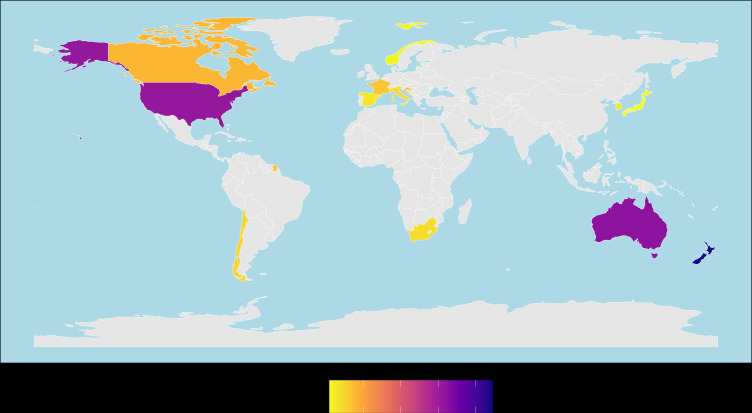
World map showing the number of study points collected in each country. The scale runs from low (yellow) to high (purple).

We found several knowledge gaps in our systematic review. As is often found in global analyses, certain countries were over-represented. The most data points came from New Zealand, Australia and the United States of America, while other countries had 20 or fewer data points (figure 3). Similarly, there were many more points for *Ecklonia* spp. and *Cystoseira* spp. than other genera (electronic supplementary material, figure S1). Therefore, it is difficult to determine if there are inherent differences between countries or kelp genera, but we found no trends in the data suggesting differences, indicating these trends may be universal (electronic supplementary material, figures S1 and S2). Metadata on kelp forest size, barren size, distance between habitats, marine protection zoning and others were missing from most studies and prevented us from exploring questions related to those categories. Many of the categories included had very small sample sizes that produced equivocal results. Larger sample sizes may generate results with higher certainty.

Several limitations of meta-analyses should be considered in this review. As we selected narrow search criteria of paired studies to reduce bias (e.g. from location, time, etc.), we found that many ecosystem functions were not well represented, and this excluded a large body of research where these functions were studied only for one ecosystem at a time. Similarly, we must acknowledge that research bias and publication bias may over-represent the significance of effects [125]. While these results, therefore, should be interpreted with caution, both the meta-analysis and supplementary review show consistent findings that kelp forests provide vastly more ecosystem services than urchin barrens.

Interestingly, many of the most cited benefits of kelp forests, primary productivity, fisheries benefits and nutrient cycling [86,93,126] were not well represented in the meta-analysis. While kelp forests are frequently described as among the most productive environments on the planet [105], no studies have compared productivity directly to urchin barrens, especially at the ecosystem level. The only taxa clearly described for fisheries were abalone and sea urchins. Notably absent from this analysis were physical, chemical or behavioural functions, such as habitat structure, environmental stabilization, predatory refugia and cultural services, which are services often attributed to kelp forests. Most studies focused on the ecological attributes of the higher profile elements of the ecosystem, namely, biodiversity, taxonomic richness and the biomass, density and productivity of kelp, sea urchins and certain fish species. Therefore, additional future studies should measure differences in physical and chemical ecosystem functions, such as pH, wave flow, sedimentation or water temperature as well as ecological interactions (feeding, breeding, etc.).

## 5. Conclusion

Our results underscore a significant ecological divergence between two alternative stable states in rocky reefs. We found kelp forests to have higher ecosystem functions and biodiversity than urchin barrens. The shift from kelp forests to urchin barrens may lead to a reduction in many ecosystem functions, many of which have a very high value to society [15,79,93]. Conversely, restoring urchin barrens to kelp forests will, on average, increase ecosystem services and benefit people. Some ecosystem attributes were higher in urchin barrens, namely those that define these habitats: urchin density, biomass and CCA, though these may not necessarily contribute to any clear services. Furthermore, this study also highlighted that many of the commonly cited benefits of kelp forests, which were apparent in our secondary literature review, were absent from the meta-analysis of paired studies. Ultimately, this study is an important step in understanding the variation between kelp forests and urchin barrens and priorities for future research. Considering the differences between the two systems will allow for informed decision-making in marine ecosystem management, particularly in a rapidly changing world with shifting baselines and altered environmental conditions.

## Data Availability

All the data and our code are freely available on Open Science Framework [127]. Supplementary material is available online [128].
